# SQUID-based ultralow-field MRI of a hyperpolarized material using signal amplification by reversible exchange

**DOI:** 10.1038/s41598-019-48827-5

**Published:** 2019-08-27

**Authors:** Seong-Joo Lee, Keunhong Jeong, Jeong Hyun Shim, Hyun Joon Lee, Sein Min, Heelim Chae, Sung Keon Namgoong, Kiwoong Kim

**Affiliations:** 1 Ultra-low Magnetic Field Team, Korea Research Institute of Standards and Science (KRISS), 267, Gajeong-ro, Yuseong-gu, Daejeon, 34113 Republic of Korea; 2 Department of Chemistry, Korea Military Academy, 574, Hwarang-ro, Nowon-gu, Seoul, 01805 Republic of Korea; 3 Department of Medical Physics, University of Science and Technology (UST), 217, Gajeong-ro, Yuseong-gu, Daejeon, 34113 Republic of Korea; 4 Department of Chemistry, Seoul Women’s University, 621, Hwarang-ro, Nowon-gu, Seoul, 01797 Republic of Korea; 5 Present Address: Electronics and Telecommunications Research Institute (ETRI), 218, Gajeong-ro, Yuseong-gu, Daejeon, 34129 Republic of Korea

**Keywords:** Magnetic resonance imaging, Chemical physics, Superconducting devices

## Abstract

The signal amplification by reversible exchange (SABRE) technique is a very promising method for increasing magnetic resonance (MR) signals. SABRE can play a particularly large role in studies with a low or ultralow magnetic field because they suffer from a low signal-to-noise ratio. In this work, we conducted real-time superconducting quantum interference device (SQUID)-based nuclear magnetic resonance (NMR)/magnetic resonance imaging (MRI) studies in a microtesla-range magnetic field using the SABRE technique after designing a bubble-separated phantom. A maximum enhancement of 2658 for ^1^H was obtained for pyridine in the SABRE-NMR experiment. A clear SABRE-enhanced MR image of the bubble-separated phantom, in which the para-hydrogen gas was bubbling at only the margin, was successfully obtained at 34.3 μT. The results show that SABRE can be successfully incorporated into an ultralow-field MRI system, which enables new SQUID-based MRI applications. SABRE can shorten the MRI operation time by more than 6 orders of magnitude and establish a firm basis for future low-field MRI applications.

## Introduction

Magnetic resonance imaging (MRI) is usually carried out in a strong magnetic field because of the Zeeman effect. Because the signal intensity depends on the population difference between nuclei in the upper and lower energy states, which is proportional to the strength of the applied magnetic field, the magnetic resonance (MR) signal intensities will be considerably higher on instruments with more powerful magnets. However, maintaining a stable strong magnetic field with a strong magnet requires great effort and cost. The magnitude of the MR signal can be enhanced by the so-called hyperpolarization process, in which a considerable number of magnetic nuclei are induced into an identical spin state. This process results in a considerable increase in the level of available signal strength due to the much greater population inequality across energy levels. Many hyperpolarization methods have been widely studied: spin-exchange optical pumping of a noble gas^1,2^, optical pumping in semiconductors^3^, chemically induced dynamic polarization^4^, and photoexcited triplet states^5^. The most common hyperpolarization technique, already in clinical trials, transfers polarization from electron polarization to neighbouring nuclei, which is known as dynamic nuclear polarization (DNP)^6^. Recently, refined dissolution DNP was optimized by hyperpolarizing electron spin and its transfer to the nuclei to adapt to high-field MRI^7,8^. However, its transfer from nuclear magnetic resonance (NMR) to MRI applications necessitates overcoming several obstacles, such as the long T_1_ time (i.e., the polarization sustaining time), efficiency, and purification of the substrate from the catalyst. In addition, the DNP method is expensive and loses a significant degree of polarization when the sample is thawed and heated to room temperature.

Alternatively, one of the most promising methods for enhancing the NMR signal strength was proposed by Bowers and Weitekamp in 1986^9^. They suggested that the addition of H_2_ molecules enriched in the para-state can result in the enhancement in NMR signals. This method was initially called para-hydrogen and synthesis allows dramatically enhanced nuclear alignment (PASADENA) and was later named para-hydrogen induced polarization (PHIP)^10^. Another hyperpolarization technique, called signal amplification by reversible exchange (SABRE), which was developed recently, is considered a significant extension of PHIP^11^. The SABRE hyperpolarization technique is relatively fast, cheap, and effective and can be successfully used to achieve a non-Boltzmann state necessary to improve the MR signal strength.

On the other hand, low-field and microtesla MRI are promising tools in several fields that can surpass the merits of high-field MRI. These techniques can avoid the potential risk of high-field MRI, and the greater relaxation contrast in low-field MRI than in high-field MRI has been proposed for the detection of tumour cells^12–17^. Furthermore, the simultaneous detection of various MRI-active nuclei^18^ is a powerful tool for obtaining important information in a short time. In addition, microtesla MRI can change the magnetic field in a short time and harness magnetic field-dependent MRI information^19^. However, the magnetic signal caused by the Zeeman effect in a microtesla-range magnetic field is extremely low. Therefore, highly sensitive sensors, such as a superconducting quantum interference device (SQUID)^20^ or an optical atomic magnetometer^21^, are required to detect the NMR/MRI signals. Because the MR signal obtained by these sensors does not have an inductive detection loss depending on frequency, one may reduce the signal loss at the ultralow field by replacing the conventional inductive coil with these sensors. In addition, to obtain a good MR image in SQUID-based microtesla MRI, a pre-polarization field (*B*_p_) is required before the MR signal from the sample is detected. Therefore, some attempts^17,22,23^ were made to obtain a strong *B*_p_, including optimizing the *B*_p_ coils and cooling these coils to reduce noise. Increasing *B*_p_ using a coil also yielded critical effects: the generation of eddy currents on the walls of a magnetically shielded room (MSR)^24,25^ and flux trapping in a pickup coil^26^. These side effects can hamper the development of microtesla MRI in the future.

The hyperpolarization technique can solve this pre-polarization problem to enable the polarization of materials with an extremely high order of magnitude. There have been several recent reports^18,22,27–30^ on this strategy. The most well-known way of achieved hyperpolarization, DNP, which is used to transfer polarization from electrons to nuclei, was harnessed to obtain a high-contrast microtesla MR image^18,22^. Alternatively, SABRE, which induces hyperpolarization on a variety of substrates via low-cost, high-throughput hyperpolarization without complex devices under normal conditions, utilizes para-hydrogen (*p*-H_2_) by forming a metal complex with some target substrates and transfers hyperpolarization from a polarized proton to a weakly bound substrate^31–33^. Polarization transfer is carried out by matching the scalar coupling/chemical shift and Zeeman energy of active nuclei in a complex structure. Because the ligands in the complex are weakly bound, the exchange of ligands continuously builds up a pool of hyperpolarization in the solution. Several MR studies with SABRE were performed at low field (47.5 mT)^34,35^ and earth’s magnetic field^36^. Moreover, Buckenmaier *et al*. reported the detection of a hyperpolarized NMR signal using SQUID-based NMR with SABRE^29^. However, despite its great potential, technically controlled investigations under low-magnetic-field conditions, especially those under ultralow-magnetic-field conditions, are lacking.

**Figure 1:**
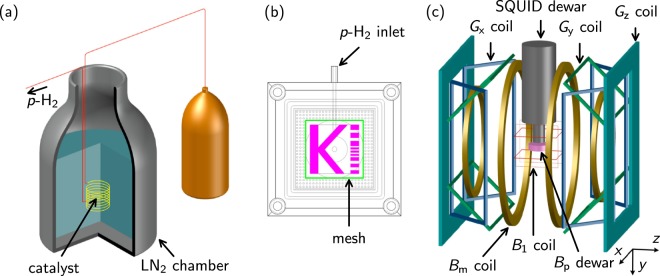
Experimental equipment for the microtesla para-hydrogen (*p*-H_2_) NMR/MRI experiments. (**a**) Schematic diagram of the *p*-H_2_ generator. Approximately 50% *p*-H_2_ was generated through a liquid-nitrogen (LN_2_) chamber including an iron oxide-based catalyst. (**b**) Schematic diagram of a 3D phantom containing a methanol solution in which pyridine as a substrate and an Ir catalyst were dissolved. The *p*-H_2_ gas entered the 3D phantom through the *p*-H_2_ inlet and many holes in the bottom and generated bubbles. A mesh was attached to prevent the generation of bubbles in the imaging region. (**c**) Experimental apparatus for the microtesla MRI experiment. As a *B*_p_ coil, a silicon-oil-cooled pancake-type coil mounted inside a *B*_p_ Dewar was used. A double Helmholtz coil was used as the *B*_m_ coil. Two pairs of square-type Helmholtz coils, aligned mutually orthogonally, and the remaining coils were used as the *B*_1_ coil and three-axis gradient coils, respectively.

Using the above information, we matched the condition for the highest SABRE polarization and designed a SABRE-based MRI phantom. This phantom can be used to obtain a real-time accumulated MR signal from a hyperpolarized proton signal of a small concentration of pyridine by continuously bubbling (Fig. 1). Therefore, in this study, we report the first SQUID-based microtesla MR image based on the para-hydrogen SABRE system. An enhancement factor of more than 2650 and a clear SABRE-derived ^1^H MR image of the phantom were obtained with 8 mT *B*_p_ at 34.3 µT.

## Results

### Magnetic field-dependent phase changes for ortho-, meta-, and para-hydrogen on pyridine

**Figure 2:**
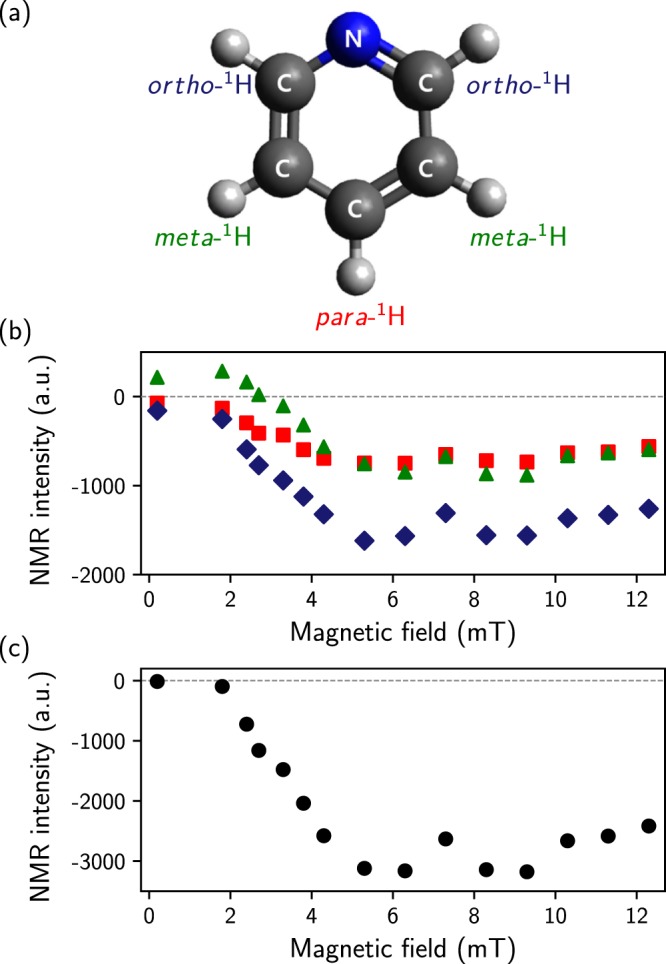
SABRE-enhanced ^1^H NMR results, measured at 1.4 T. (**a**) Structure of pyridine with labeled protons. (**b**) NMR intensity of each attached proton versus the applied magnetic field for hyperpolarization. (**c**) Merged NMR intensity as a function of the applied magnetic field. The data were obtained by summing three types of hydrogen peaks on pyridine in each magnetic field. The dashed line is for visual guidance.

SABRE polarization is sensitive to an external field, which induces hyperpolarization in a weak magnetic field. Furthermore, quantum mechanically, *p*-H_2_ gives only the singlet state, which is an anti-aligned hyperpolarized spin state of the proton, when it induces polarization transfer; therefore, its phase in polarization may depend on the external magnetic field due to the polarization transfer mechanism, which is related to the chemical shift and scalar coupling of the active SABRE complex^37–39^. The polarized nuclear spin states from *p*-H_2_ are more complex than those from polarization at thermal equilibrium. Both of the above factors present a challenge in obtaining polarization in a low field. The challenge arises from the fact that *p*-H_2_-derived ^1^H NMR signals often have an anti-phase character, which results in significant signal cancellation if resonances are not resolved. Therefore, we observed magnetic field-dependent phase changes for ortho-, meta-, and para-hydrogen on pyridine (Fig. 2) and optimized the external field for the most efficient in-phase polarization transfer on all protons in pyridine. As suggested in other reports^37,40^, polarization of the protons in all positions (ortho-, meta-, and para-) on pyridine was maximized in the range from 5 to 9 mT (Fig. 2(b,c)). The phase changes are due to the mismatch between the Zeeman effect and the J-coupling when SABRE chemical exchange is conducted in a lower magnetic field, e.g., earth’s magnetic field of approximately 50 µT, as proposed in previous studies^37–39^.

### Dependence of the enhancement factor on the *B*_p_ strength and the *B*_p_ time

**Figure 3:**
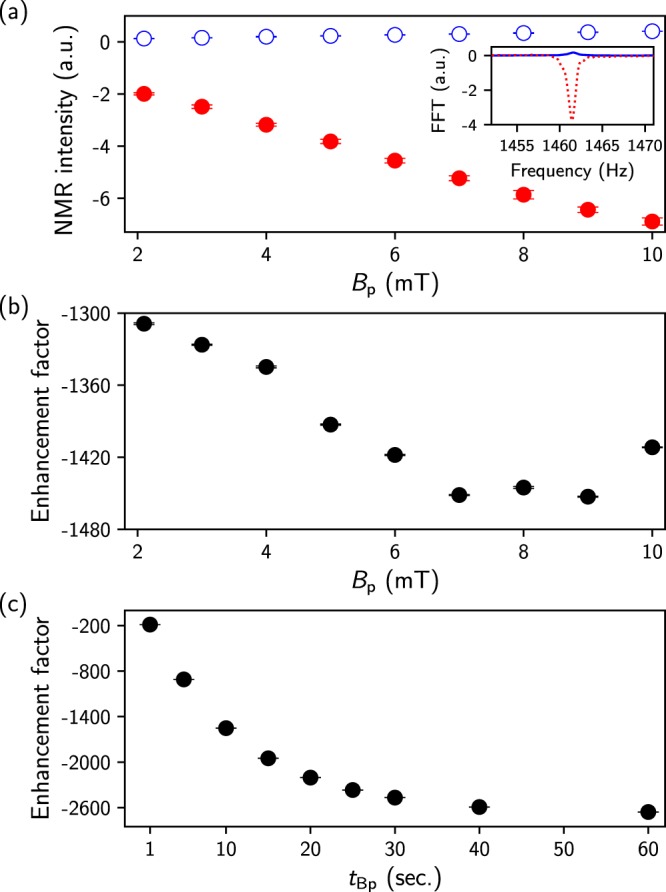
SABRE-enhanced ^1^H NMR results. (**a**) NMR intensity as a function of the *B*_p_ strength obtained with a fixed time (*t*_Bp_ = 10 s). The inset shows the FFT spectra with (dashed line) and without (solid line) *p*-H_2_ flowing at 8 mT *B*_p_. (**b**) Enhancement factor versus *B*_p_. (**c**) Enhancement factor as a function of the *B*_p_ time (*t*_Bp_) obtained at 8 mT *B*_p_. All data were measured four times at each point. Error bars represent the standard error of repeated measurements. NMR intensities were obtained with a real spectrum area and phase correction at each data point. The enhancement factors were calculated with the additional considerations of the volume and nuclear spin ratios of pyridine and methanol.

The microtesla NMR experiment was performed to investigate the effect of the SABRE-enhanced ^1^H NMR signal (i.e., the pyridine signal) on the external field and the application time of this field. The dependence of the NMR signal on *B*_p_ is displayed in Fig. 3(a). Because the chemical shifts of the ortho-, meta-, and para-hydrogen on pyridine, including the methanol peak, cannot be distinguished in the microtesla range, all peaks are mixed in the presence of a *p*-H_2_ gas stream. On the other hand, when the *p*-H_2_ flux is absent, the NMR signal of methanol is the main contributor to the measurable proton signal because the proton population difference after the addition of pyridine is relatively small. Since the phase of the SABRE-enhanced NMR signal, compared to that of the reference signal, was inverted, as shown in the inset of Fig. 3(a), the SABRE-enhanced NMR signal intensity (closed circles) and the reference signal intensity (open circles) increase in the negative and positive direction, respectively, as *B*_p_ increases. The inset shows the FFT spectra with (dashed line) and without (solid line) *p*-H_2_ flowing at 8 mT *B*_p_. Therefore, the enhancement factor (*E*_SABRE_) can be defined as follows:

1 $${E}_{{\rm{SABRE}}}=({S}_{{\rm{SABRE}}}/S-1)\alpha \beta $$

where *S*_SABRE_ is the SABRE-enhanced NMR signal, *S* is the reference NMR signal of all protons when there is no *p*-H_2_ gas flow, and *α* (=99) and *β* (=4/5) are the volume and nuclear spin ratios of the pyridine and methanol, respectively. The dependence of *E*_SABRE_ on *B*_p_ is shown in Fig. 3(b). *E*_SABRE_ increases with increasing *B*_p_ and then reaches a maximum value around a *B*_p_ of 7 ~ 9 mT. The overall trend in *E*_SABRE_ is quite consistent with the result for the high-field experiment shown in Fig. 2(c). The *E*_SABRE_ was also obtained as a function of *B*_p_ time (*t*_Bp_) at 8 mT *B*_p_ (Fig. 3(c)). In our experiments, *E*_SABRE_ began to converge to a maximum value for a *B*_p_ time above 30 s and increased up to approximately 2658 for *t*_Bp_ = 60 s.

### SABRE-enhanced ^1^H MR image

**Figure 4:**
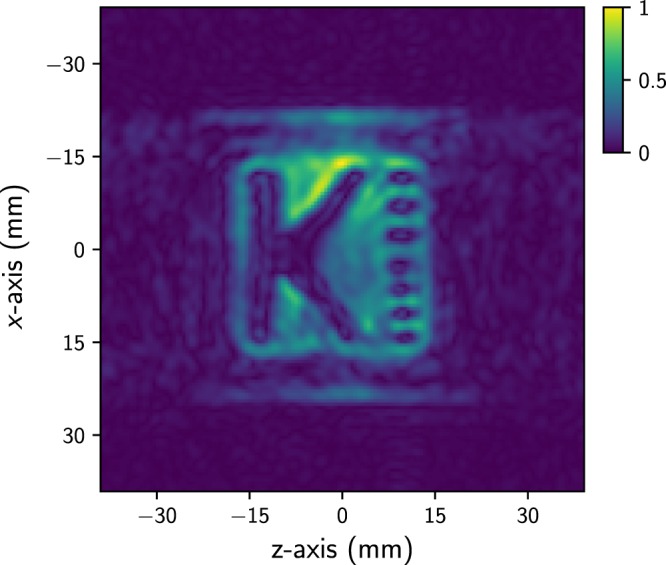
SABRE-derived ^1^H MR image obtained at 34.3 µT. The MR image was obtained in 4 iterations. The total experimental time for obtaining the MR image was approximately 47 min.

The SABRE-derived ^1^H MR image, obtained with 8 mT *B*_p_ at 34.3 µT, is displayed in Fig. 4. As more methanol evaporated with increasing total imaging time, the MR experiment was performed while accounting for the total imaging time and image resolution. The following experimental parameters were used: *t*_Bp_ = 13 s, *t*_pe_ = 0.12 s, *t*_acq_ = 0.3 s, ${G}_{{\rm{x}}}=|\pm {G}_{{\rm{z}},{\rm{\max }}}|=0.6$µT/cm, 49 phase encoding steps (Δ*G*_z_ = 0.025 µT/cm), and 4 iterations. Therefore, the resolution, estimated from the experimental parameters, was approximately 1.6 × 1.6 mm^2^. On the other hand, the enhancement factor in the MR experiment was more than 1800 at a given *t*_Bp_(=13 s), which was estimated by interpolation of the data in Fig. 3(c). Because the MR signal obtained by the SQUID sensor is proportional to the magnetic field, including the measurement field (*B*_m_) and *B*_p_, it is possible to predict a polarization enhancement of approximately 420,000-fold by comparison with the thermal polarization at 34.3 µT *B*_m_. In the MR image, the outside regions, denoted as the “mesh” in Fig. 1(b), were only faintly visible because of the bubbles of *p*-H_2_ gas. In contrast, the inside regions were reasonably clearly imaged. Among the nine rectangular blocks located on the right side of the phantom, the three rectangular blocks located in the middle could not be distinguished from each other individually on the MR image because these patterns were smaller than the imaging resolution.

## Discussion

We performed high-field NMR and microtesla NMR/MRI experiments. From the high-field NMR results, it was confirmed that all protons of pyridine had in-phase polarization in an external field greater than approximately 3 mT. An enhancement factor exceeding 2650, compared with the NMR intensity of the methanol solution, was obtained with 8 mT *B*_p_ at 34.3 µT. An enhanced MR image of the bubble-separated phantom was obtained using the SABRE technique in the ultralow field. In that phantom, the bubbles are formed at only the margin, and the hyperpolarized pyridine from the bubbling region is diffused into the imaging region. The advantage of the experimental approach introduced in this study is that the relaxation time is negligible because hyperpolarization generation and MR imaging can be performed at the same place at the same time. One of the breakthroughs that must be made in conventional MRI is a shortening of the operation time, which is usually lengthened by increasing the scan time to obtain a sufficient amount of signal-to-noise^41–43^. An enhancement factor on the order of a few thousands in real-time NMR/MRI was achieved in the ultralow field, even though the actual level of polarization was on the order of 0.00007%; this value was estimated from the equation *E*_SABRE_ × tanh(*γħB*_p_/2*k*_B_*T*), in which *γ* is the gyromagnetic ratio of a proton, *T* is the temperature, and ℏ and *k*_B_ are Planck’s constant and the Boltzmann constant, respectively. This result means that it is possible to shorten the scan time by more than 6 orders of magnitude. After induction of hyperpolarization on biomaterials near MRI devices under normal conditions, the introduction of a hyperpolarized material into the patient would enable a short-time MRI operation. Until now, however, *in vivo* imaging by the SABRE-hyperpolarization technique has not been reported. Furthermore, additional sensitivity enhancement would be possible with, for example, more hyperpolarized para-hydrogen, deuterated substances, or polarization transfer to ^13^C or ^15^N with a long-lived nuclear spin state^44–46^. Our result, based on this concept, may open up a new field of application of low-field MRI. This work showed the possibility of obtaining a hyperpolarized SQUID-based microtesla MR image based on the SABRE system, which means that any imaging studies using the SABRE system are extended to further advanced studies, especially low-field hyperpolarized MRI. Furthermore, this method led to polarization enhancement by more than 420,000-fold in hyperpolarized proton MRI. This study has established a firm basis for a future low-field MRI system, which holds many advantages over a high-field MRI system. Furthermore, detailed and large-scale research on the development of new SABRE systems and hyperpolarization in biomaterials, such as SABRE-Relay^47^ and SABRE in shield enables alignment transfer to heteronuclei (SABRE-SHEATH)^48^, would reduce the time required for the actual application of SQUID-based microtesla MRI in human and magnetic resonance spectroscopy. These ongoing integrated studies will open up a new area of MRI applications that will allow the detection of many useful materials, tumours, and even pathogens in humans by microtesla MR imaging.

## Methods

### Chemistry

All chemicals were purchased from Sigma-Aldrich and used without further purification unless otherwise indicated. All SABRE experiments were performed with the Ir catalyst [Ir(COD)(IMes)(Cl)] (2 mM) in methanol (approximately 20 mL for SQUID and 0.7 mL for high-field NMR) without degassing^49^. A pyridine solution of approximately 124 mM (10 µL in 1 mL of methanol) was hyperpolarized by polarization transfer.

### Para-hydrogen

Approximately 50% *p*-H_2_ was generated by the catalytic conversion at liquid-nitrogen temperature using a home-built *p*-H_2_ generator (Fig. 1(a)). Hydrogen gas (a mixture of spin isomers of hydrogen, i.e., ortho-hydrogen and para-hydrogen) was passed through a heat exchanger filled with a FeO(OH) catalyst in a liquid-nitrogen Dewar.

### High-field NMR

A Spinsolve Ultra (Magritek) spectrometer operating at 60 MHz was used to obtain hyperpolarized ^1^H NMR spectra of pyridine. The instrument was shimmed on 5% D_2_O using an automated algorithm, and the NMR signals were tested with pyridine in benzene. A mixture of pyridine (10 µL) and methanol (1 mL) was added to a 5 mm NMR tube after lowering the oxygen level in the solution by bubbling pure nitrogen gas for approximately 2 min. A home-built solenoid coil was set up to induce the designed magnetic field. Each designed magnetic field was formed to study the magnetic field-dependent phase change. In each magnetic field, the generated *p*-H_2_ was bubbled into a 5 mm NMR tube for 30 s at 23 °C and 1 atm and transferred into the spectrometer (<1 s). Spectra were obtained at 60 MHz and 23 °C in one scan using a *π*/2 proton pulse. The NMR data were processed and analysed using Mnova software (Mestrelab Research, S.L.).

### Experimental setup for microtesla NMR/MRI

Figure 1(b) shows the 3D-printed *p*-H_2_ phantom with inner dimensions of 50 (L) × 50 (W) × 21.3 (H) mm^3^. The *p*-H_2_ phantom contained pyridine and an Ir catalyst dissolved in a methanol solution. The thickness of the character “K” was approximately 5 mm. Nine rectangular blocks were placed on the right side of the phantom: (top) three blocks with dimensions of 5 (L) × 3 (W) mm^2^ and 2 mm spacing, (middle) three blocks with dimensions of 5 (L) × 1 (W) mm^2^ and 1 mm spacing, and (bottom) three blocks with dimensions of 5 (L) × 2 (W) mm^2^ and 2 mm spacing. Because the *p*-H_2_ gas entered the 3D phantom through a *p*-H_2_ inlet and many holes in the bottom and generated bubbles, a mesh was attached to prevent the generation of bubbles in the imaging region.

Figure 1(c) shows the experimental setup for SQUID-based NMR/MRI experiments installed inside the MSR. A dc-SQUID connected with a second-order gradiometric pickup coil placed inside a liquid-helium SQUID Dewar was used to detect the NMR/MRI signal. *B*_m_ and *B*_p_ were generated using a double Helmholtz coil and a pancake-type coil cooled with silicon oil that was mounted inside the *B*_p_ Dewar, respectively. A circularly polarized nutation pulse (*B*_1_)^50^ was used to avoid the Bloch-Siegert effect in the microtesla region. Two pairs of square-type Helmholtz coils, aligned orthogonally to each other, were used to create a circularly polarized *π*/2 *B*_1_. Three-axis gradient fields (*G*_x_, *G*_y_, and *G*_z_) were generated using the rest coil sets. Because the *p*-H_2_ generator shown in Fig. 1(a) was placed outside the MSR, the 3D phantom, mounted between the SQUID and *B*_p_ Dewars, and the *p*-H_2_ generator were connected using an 8.5 m tube. During the NMR and MRI experiments, *p*-H_2_ gas flowed continuously at a rate of 70 mL/min and bubbled into the reaction mixture inside the 3D phantom at room temperature.

### Pulse sequence for microtesla NMR/MRI

**Figure 5:**
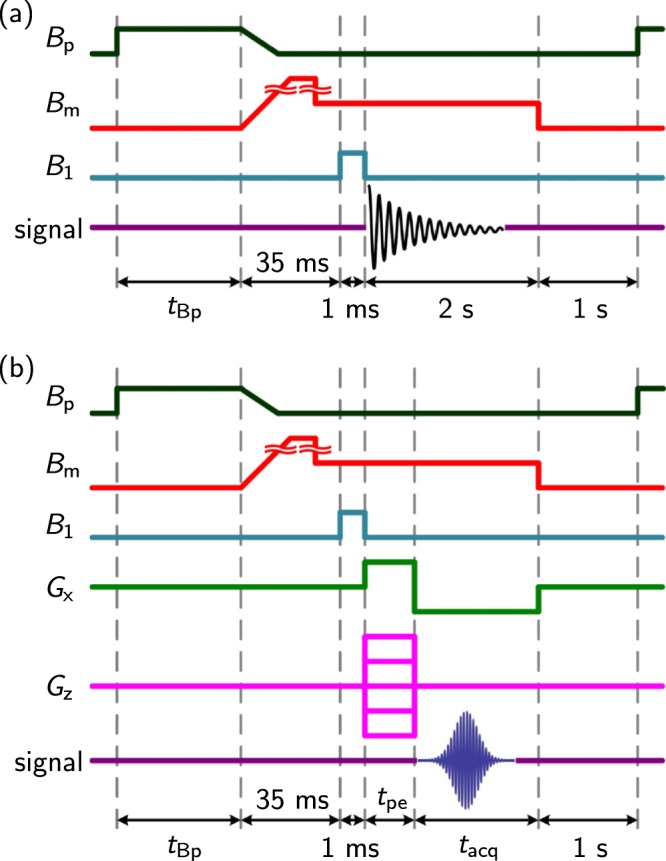
Pulse sequences for the microtesla *p*-H_2_ NMR/MRI experiments. (**a**) Illustration of the free-precession-decay pulse sequence. (**b**) Illustration of the gradient echo pulse sequence.

Figure 5 shows the NMR and MRI pulse sequences used in this study. Because an external field on the order of millitesla is necessary to create SABRE polarization, *B*_p_ was applied during the time *t*_Bp_. In this study, the variable range of the *B*_p_ strength, measured at the centre of the top plate of the *B*_p_ Dewar, was approximately 2.1 to 10 mT. Because the axes of the coils *B*_p_ and *B*_m_ are perpendicular to each other, the nuclear spins were initially aligned parallel to the direction of *B*_p_. To rotate the spins in the direction of *B*_m_, an adiabatic pulse^23^ (i.e., an adiabatic change in *B*_p_ and *B*_m_ followed by a non-adiabatic change in *B*_m_) was applied for 35 ms. *B*_m_ strengths of 106.6 and 34.3 µT were used to satisfy the adiabatic condition and detect the NMR/MRI signal, respectively. After the application of circularly polarized *π*/2 *B*_1_, the NMR signal or the MRI signal following the additional gradient echo pulse can be measured using the SQUID sensor. During the MRI experiments, *G*_x_ and *G*_z_ were used as the frequency- and phase-encoding gradients, respectively.

## Data Availability

The relevant data supporting the findings of this study are available within the paper or are available from the corresponding author, K.K., upon reasonable request.
